# *Radix Hedysari* polysaccharide suppresses lipid metabolism dysfunction in a rat model of non-alcoholic fatty liver disease via adenosine monophosphate-activated protein kinase pathway activation

**DOI:** 10.3892/mmr.2014.2327

**Published:** 2014-06-13

**Authors:** WEI-MING SUN, YU-PING WANG, YONG-QIANG DUAN, HONG-XIA SHANG, WEI-DONG CHENG

**Affiliations:** 1 Institute of Integrated Chinese and Western Medicine, School of Basic Medical Sciences, Lanzhou University, Lanzhou, Gansu 730000, P.R. China; 2 Department of Endocrinology, The First Hospital of Lanzhou University, Lanzhou, Gansu 730000, P.R. China; 3 Key Laboratory for Gastrointestinal Diseases of Gansu Province, Lanzhou University, Lanzhou, Gansu 730000, P.R. China; 4 Department of Gastroenterology, The First Hospital of Lanzhou University, Lanzhou, Gansu 730000, P.R. China; 5 Department of Basic Courses, Gansu College of Traditional Chinese Medicine, Lanzhou, Gansu 730000, P.R. China; 6 School of Traditional Chinese Medicine, Southern Medical University, Guangzhou, Guangdong 510515, P.R. China

**Keywords:** *Radix Hedysari*, polysaccharide, lipid metabolism, non-alcoholic fatty liver disease, adenosine monophosphate-activated protein kinase pathway

## Abstract

Oxidative stress and excess hepatic lipid accumulation contribute to non-alcoholic fatty liver disease. *Radix Hedysari* polysaccharides (RHP) have attracted interest due to their antioxidant properties and immunomodulatory effects. However, the effect of RHP on hepatic lipid metabolism remains to be elucidated. In the present study, the response of Sprague-Dawley rat livers to a high-fat diet and RHP treatment was investigated by evaluating body weight, liver histology, hepatic lipid content, adenosine monophosphate-activated protein kinase (AMPK) activity and lipid metabolism gene transcriptional profiles. The present study demonstrated that RHP ameliorated lipid metabolism disorders, regulated hepatic lipid content, improved liver inflammation and damage, activated AMPK via phosphorylation, upregulated peroxisome proliferator-activated receptor α and downregulated the mRNA expression of sterol regulatory element binding protein-1c in rat livers, which reduced lipogenesis and increased lipolysis. Taken together, these results suggested that RHP effectively ameliorates lipid metabolism disorders in rat livers; thus, RHP may be a potential therapeutic agent in the prevention of hepatic steatosis.

## Introduction

Non-alcoholic fatty liver disease (NAFLD) is a major cause of chronic liver disease in numerous countries and has become an international public health threat. NAFLD represents a wide spectrum of liver diseases ranging from simple steatosis to nonalcoholic steatohepatitis (NASH). While simple steatosis is usually non-progressive, patients with NASH may develop cirrhosis and associated complications, including liver failure and hepatocellular carcinoma (1). In addition to its liver-related complications, NAFLD is increasingly considered to be the hepatic manifestation of metabolic syndrome and is strongly associated with obesity, diabetes mellitus and cardiovascular disease (2).

The pathogenesis of NAFLD remains to be elucidated but a ‘two-hit’ mechanism has been widely accepted to describe its development. The first ‘hit’ is hepatic steatosis, which is characterized by excess hepatic lipid accumulation caused by increased *de novo* lipogenesis and adipose tissue lipolysis. Hepatic steatosis is important in the initiation and development of NAFLD. The second ‘hit’ is oxidative stress and inflammatory response, which induce hepatocellular injury and fibrosis. Intrahepatic triglyceride level can be reduced following diet-induced weight loss and regular physical activity. Similarly, NAFLD-associated metabolic derangements can be restored by lifestyle modification as well. However, there are no FDA-approved treatments for this problem (3). Previous studies have focused on identifying the active ingredients of natural products or herbal extracts that can inhibit adipogenesis and induce adipocyte apoptosis (4,5).

Radix Hedysari (RH) is the dried root of *Hedysarum polybotrys* Hand-Mazz and has been widely used in Chinese traditional medicine for its tonifying Qi, diuretic and circulatory effects. *Radix Hedysari* polysaccharide (RHP) is the main bioactive ingredient of RH, and possesses several pharmacological activities, including antitumor, anti-inflammatory, antioxidant and immunomodulatory effects (6,7). Previous studies have also suggested that RHP may have hypoglycemic and hypolipidemic properties and may improve insulin resistance (8), however, the effect of RHP on NAFLD remains to be elucidated.

The present study aimed to examine the anti-hyperlipidemic and hepatoprotective effects of RHP in a high-fat diet (HFD)-induced rat model of NAFLD, and the possible mechanisms underlying these effects were evaluated.

## Materials and methods

### RHP preparation

RH roots were gathered from the Wudu County mountain region (Gansu, China). The RHP was purified by the Institute of Combined Traditional Chinese and Western Medicine, School of Basic Medical Science at Lanzhou University (Lanzhou, China) (6,9). The polysaccharide content was 97.3%.

### Animals

Male Sprague-Dawley rats (Gansu University of Traditional Chinese Medicine, Lanzhou, China) weighing 160±20 g were fed in a particular pathogen-free laboratory environment with free access to standard pellet chow and sterile water and were maintained under a 12 h light/dark circadian cycle. Food intake and rat body weight were monitored weekly. The study was approved by the Ethics Committee of Gansu College of Traditional Chinese Medicine (Lanzhou, China) and appropriate measures were taken to reduce any pain or discomfort of the rats.

### Experimental design

Following the acclimatization period, 42 rats were randomly divided into two groups. Of these, 10 animals were fed a regular diet (RD) and 32 animals were fed a HFD for 8 weeks. The diets were analyzed for nutritional content as indicated in Table I. The established rat model was confirmed by histopathological examination in two rats and the 30 remaining rats were separated into three random groups (10 animals/group).

The first group (RD) received a regular diet and an oral gavage of physiological saline solution. The second group (HFD) received a HFD and an oral gavage of physiological saline solution. The third group (HFD + RHP_50_) and the fourth group (HFD + RHP_150_) received a HFD and were administered with oral gavages of 50 and 150 mg/kg RHP liquid, respectively. All rats were treated once daily for 8 weeks. Every effort was made to minimize any animal suffering and to reduce the number of animals used based on the Chinese Guidelines for the Care and Use of Laboratory Animals.

### Blood and tissue collection

At the end of the treatment period, food was removed for 12 h and drinking water was allowed ad libitum. Rats were then anesthetized with sodium pentobarbital (60 mg/kg, i.p.; Harbin Pharmaceutical Group Co., Ltd., Harbin, China). Blood samples were collected from the femoral artery and were immediately placed into ice-chilled silicon disposable glass tubes (Shanghai Showbio Biotech, Inc., Shanghai, China) and allowed to stand for 30 min. Blood samples were centrifuged at 2,500 × g for 15 min at 4°C to obtain serum, which was stored in aliquots at −80°C prior to analysis. Liver tissues were dissected, weighed, frozen in liquid nitrogen and stored at −80°C prior to use.

### Liver morphological analysis and lipid staining

Hematoxylin and eosin (H&E) staining was performed on liver sections according to standard instructions (10). The livers were fixed, dehydrated, dipped in paraffin (Shanghai Showbio Biotech, Inc.), sectioned (8 μm) and stained with H&E. Slides were examined using a DP70 light microscope (Olympus, Tokyo, Japan).

To observe hepatic lipid accumulation, frozen liver tissue sections were stained with Oil Red O (ORO; Shanghai Showbio Biotech, Inc.) in accordance with a previously described method (11). Briefly, liver tissues were cryosectioned (6 μm thick), fixed in 10% formalin solution (Sinopharm Chemical Reagent Co., Ltd., Shanghai, China) at room temperature for 10 min and dipped in 60% isopropanol (Sinopharm Chemical Reagent Co., Ltd.) for 3 min. The slides were then immersed in 1% ORO solution for 10 min and washed in 60% isopropanol followed by distilled water. The slides were counterstained with Mayer’s hematoxylin and were mounted onto glycerin gelatin (Shanghai Showbio Biotech, Inc.).

### Biochemical parameters of liver function

An automatic biochemical analyzer (Sysmex Chemix 180; Sysmex Corp., Kobe, Japan) was used to determine the serum alanine aminotransferase (ALT), aspartate aminotransferase (AST), high-density lipoprotein cholesterol (HDL-C), low-density lipoprotein cholesterol (LDL-C), total cholesterol (TC) and triglyceride (TG) concentrations, according to the manufacturer’s instructions.

### Hepatic TG content measurement

To determine hepatic TG content, frozen liver tissue was homogenized in phosphate-buffered saline and methanol (Sinopharm Chemical Reagent Co., Ltd.) was added to the lysate. Lipids were extracted according to the method of Bligh and Dyer (12), and TG content was detected using a TG kit (Abcam, Cambridge, UK).

### RNA extraction and reverse transcription

Total RNA was extracted from the liver tissues using TRIzol^®^ reagent (Invitrogen Life Technologies, Carlsbad, CA, USA) according to the manufacturer’s instructions. The concentration and quantity of isolated total RNA were measured using a Nanodrop Spectrophotometer (ND-1000; NanoDrop Technologies, Inc., Wilmington, DE, USA). Reverse transcription was performed in a 25 μl reaction volume using 2 μg RNA with moloney murine leukemia virus reverse transcriptase (Promega Corporation, Madison, WI, USA).

### Semi-quantitative and quantitative polymerase chain reaction (qPCR)

Semi-quantitative RT-PCR and qPCR were performed on a Thermal Cycler Dice™ Detection System with SYBR green dye (Takara Bio, Inc., Shiga, Japan) according to the method described previously (13). The β-actin gene was used as a reference for the normalization of data. The primers used in the present study are presented in Table II.

### Western blot analysis

Western blot analysis was performed according to the method described previously (14). Total protein (50 μg) was separated onto 8% sodium dodecyl sulfate-polyacrylamide minigels and transferred onto Hybond-C nitrocellulose membranes (Amersham Life Science, Buckinghamshire, UK). Following blocking with PBS containing 5% nonfat milk, the membrane was incubated with rabbit monoclonal antibodies specific for rat adenosine monophosphate-activated protein kinase α (AMPKα), phosphorylated-AMPKα (p-AMPKAα; Thr172), acetyl-CoA carboxylase (ACC), rabbit polyclonal antibody specific for rat phosphor-ACC (Ser79; 1:1,000; Cell Signaling Technology, Inc., Beverly, MA, USA), and mouse monoclonal antibody specific for rat β-actin (1:1,000; Santa Cruz Biotechnology, Inc., Santa Cruz, CA, USA) at room temperature for 2 h or at 4°C overnight, followed by incubation with IRDye 800CW or 680Rd goat anti-rabbit or anti-mouse secondary antibodies (1:10,000; Li-COR Biosciences, Lincoln, NE, USA) at room temperature for 30 min. β-actin served as a control for sample loading and integrity. The sample bands were revealed using the Odyssey Infrared Imaging System (Li-COR Biosciences).

### Statistical analysis

All data were obtained from at least three separate experiments and are expressed as the mean ± standard deviation from 10 rats. The statistical significance was evaluated using one-way analysis of variance followed by a Newman-Keuls post-hoc test for multiple comparisons (GraphPad Software, Inc., San Diego, CA, USA). P<0.05 was considered to indicate a statistically significant difference.

## Results

### RHP ameliorates lipid metabolism disorders

**Figure 1:**
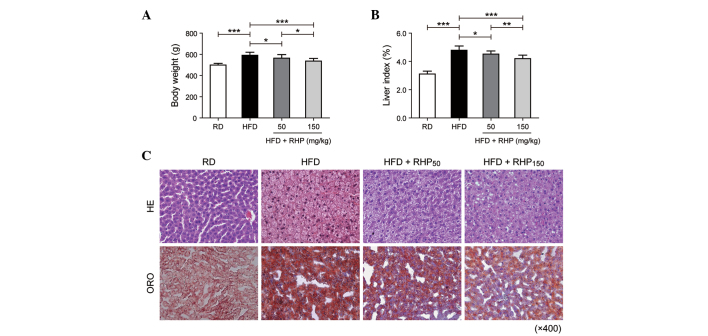
Effects of RHP administration on body and liver weight and lipid accumulation in rats. (A) Changes in body weight in the four groups. (B) Changes in liver index in the four groups. (C) Histological analysis of liver tissues (magnification, ×400). Values are expressed as the mean ± standard deviation (n=10 rats/group). All experiments were repeated at least three times to confirm results. ^*^P<0.05, ^**^P<0.01, ^***^P<0.001 between the two indicated groups. HFD, high-fat diet; RHP, *Radix Hedysari* polysaccharide; RD, regular diet; HE, hematoxylin and eosin; ORO, Oil Red O.

A HFD was fed to rats to establish an NAFLD model and the phenotypes were observed following simultaneous RHP administration. At the beginning of the investigation, the body weights of the rats in each group were not significantly different (P>0.05). At week 16, the body weights and the liver index (liver weight (g) / 100 g body weight) of the HFD group had increased (P<0.001; Fig. 1A and B) compared with the RD group. Compared with the HFD group, the intervention group body weights and liver index (HFD + RHP_50_ and HFD + RHP_150_) had markedly decreased (P<0.05, P<0.001; Fig. 1A and B).

When liver histology was evaluated by H&E staining, RD rat tissue exhibited well-arranged hepatic cords, cells with round and central nuclei, a lobular structure and an array of wheel-shaped cells along the centrilobular vein. However, in the HFD group, lipid droplets were observed in the liver sections (Fig. 1C). The accumulated lipids in rat liver tissues obtained from each experimental group were detected as red spots on histological analysis using ORO staining. ORO staining of liver sections confirmed that rats in the HFD group had an increased accumulation of hepatic lipids compared with the RD group. Lipid droplet volumes and quantities were reduced by simultaneous RHP administration.

These findings demonstrated that feeding a HFD to rats was able to successfully establish a model of disordered lipid metabolism. Furthermore, RHP intervention improved this lipid metabolism disturbance.

### RHP regulates the concentration of serum TC, TG, LDL-C, and HDL-C as well as the hepatic TG content

**Figure 2:**
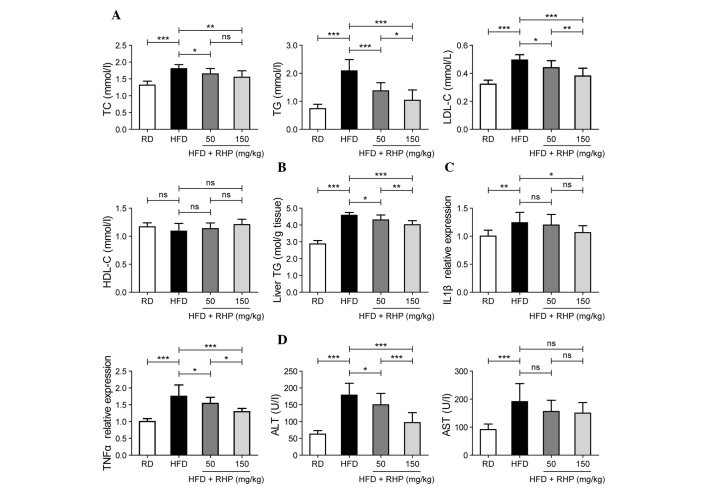
Effects of RHP administration on lipid metabolism parameters, inflammation and damage. (A) Evaluation of serum TC, TG, LDL-C and HDL-C contents in the four rat groups. (B) Evaluation of liver TG content in the four rat groups. (C) Evaluation of liver IL-1β and TNF-α mRNA expression levels in the four rat groups. (D) Evaluation of serum ALT and AST concentrations. Values are expressed as the mean ± standard deviation (n=10 rats/group). All experiments were repeated at least three times to confirm results. ^*^P<0.05, ^**^P<0.01, ^***^P<0.001 between the two indicated groups; ns indicates no statistical significance. RHP, *Radix Hedysari* polysaccharide; HFD, high-fat diet; TC, total cholesterol; TG, triglyceride; LDL-C, low-density lipoprotein cholesterol; HDL-C, high-density lipoprotein cholesterol. IL-1β, interleukin-1β; TNF-α, tumor necrosis factor-α; ALT, alanine aminotransferase; AST, aspartate aminotransferase.

Selected lipid metabolism parameters were detected in the four experimental groups. At the end of the 16th week, significant increases in serum TC, TG and LDL-C levels (P<0.001; Fig. 2A) and hepatic TG content were identified in the HFD group compared with the RD group (P<0.001; Fig. 2B), whereas HDL-C levels were unaltered (P>0.05; Fig. 2A). Significant decreases in serum TC, TG and LDL-C levels and hepatic TG content were identified in the RHP intervention groups (P<0.05; Fig. 2A and B). No significant difference in serum HDL-C levels was identified following RHP administration (P>0.05; Fig. 2A). Notably, the majority of lipid metabolism parameters exhibited a dose-effect association between these two intervention groups (P<0.05; Fig. 2A and B).

These findings also suggested that lipid metabolism disorders were present in the rat model and that RHP interventions were able to correct them.

### RHP treatment improves liver inflammation and damage

In addition to hepatic lipid accumulation, inflammation and liver cell damage also occurred frequently. As a result of the fatty liver and inflammatory condition, the mRNA expression levels of interleukin-1β (IL-1β) and tumor necrosis factor-α (TNF-α) were evaluated as markers of liver inflammation. In HFD-fed rat livers, the mRNA expression of IL-1β and TNF-α significantly increased (P<0.01; Fig. 2C). RHP administration to HFD-fed rats prevented the upregulation of IL-1β and TNF-α expression (P<0.05). Compared with the RD group, HFD-fed rats were characterized by significantly higher serum ALT and AST activities (P<0.001; Fig. 2D). However, following RHP administration, ALT activity declined (P<0.05). Although AST activity was also reduced in the two RHP groups, no significant changes were observed compared with the HFD group. Dose-effect associations in TNF-α expression and ALT activity levels were also identified between these two intervention groups (P<0.05; Fig. 2C and D).

These data revealed that a HFD caused hepatic inflammation and hepatocellular damage, which was alleviated by continuous RHP administration.

### RHP activates AMPK and regulates transcription factor expression

**Figure 3:**
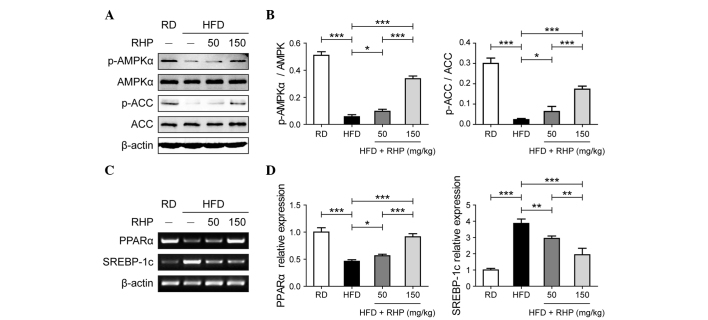
Effects of RHP treatment on AMPK signaling and transcription factor mRNA expression in rat livers. (A) Western blot analysis of RHP-induced AMPK and ACC phosphorylation in rat livers. (B) Relative protein band quantification was performed by optical density scanning of (A). (C and D) mRNA expression of transcription factors PPARα and SREBP-1c was assessed (panels C and D were obtained from semi-quantitative-PCR and quantitative PCR, respectively). All experiments were repeated at least three times to confirm results. ^*^P<0.05, ^**^P<0.01, ^***^P<0.001 between the two indicated groups. RD, regular diet; HFD, high-fat diet; RHP, *Radix Hedysari* polysaccharide; p-AMPKα, phosphorylated-adenosine monophosphate-activated protein kinase α; p-ACC, phospho acetyl-CoA carboxylase; PPARα, peroxisome proliferator-activated receptor α; SSREBP-1c, sterol regulatory element binding protein-1c; PCR, polymerase chain reaction.

The above-mentioned results obtained from the phenotypic experiment revealed that RHP treatment regulated lipid metabolism and alleviated hepatocellular inflammation and damage. The present study subsequently aimed to determine the mechanisms by which RHP treatment achieved this effect. AMPK signaling is an important pathway regulating glycolipid metabolism in hepatocytes. A decrease in the levels of p-AMPK in HFD-fed rat livers compared with RD-treated rats was observed (P<0.001; Fig. 3A and B). In rats treated with different doses of RHP, increased levels of p-AMPK were observed compared with HFD-fed rats (P<0.05). ACC phosphorylation, the activity of an important rate-limiting enzyme in lipogenesis, exhibited the same trend as p-AMPK (P<0.05; Fig. 3A and B). Relative mRNA expression levels of peroxisome proliferator-activated receptor α (PPARα) and sterol regulatory element binding protein-1c (SREBP-1c), which are transcription factors downstream of AMPK, were assessed in rat livers via semi-qPCR (Fig. 3C) and qPCR (Fig. 3D), respectively. The HFD-fed rat livers exhibited a significant decrease in PPARα mRNA expression compared with the RD rats (P<0.001) and RHP administration in HFD rats increased the mRNA expression of PPARα (P<0.05). By contrast, the expression of SREBP-1c significantly increased in the HFD group (P<0.001) and reduced following administration of RHP (P<0.01). Therefore, these data supported the hypothesis that RHP activated AMPK and regulated the expression of the downstream transcription factors PPARα and SREBP-1c.

### RHP affects lipid metabolism gene expression

**Figure 4:**
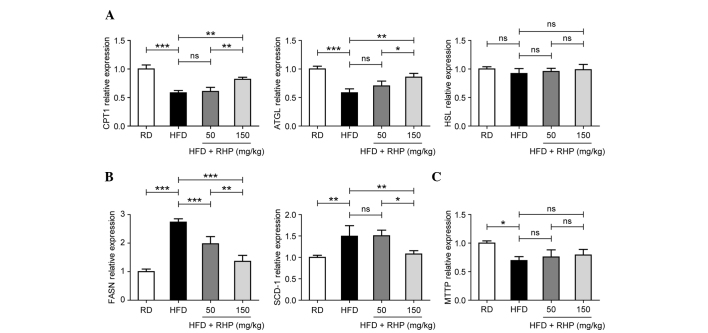
Effects of RHP administration on lipid metabolism-related gene mRNA expression in rat livers. (A) Expression of hepatic lipogenesis, (B) lipolysis and (C) lipid transport genes from the four rat groups. Data are expressed as the mean ± standard deviation (n=10 rats/group). All experiments were repeated at least three times to confirm results. ^*^P<0.05, ^**^P<0.01, ^***^P<0.001 between the two indicated groups; ns indicates no statistical significance. HFD, high-fat diet; RD, regular diet; RHP, *Radix Hedysari* polysaccharide; CPT1, carnitine palmitoyltransferase I; ATGL, adipose triglyceride lipase; HSL, hormone-sensitive lipase; FASN, fatty acid synthase; SCD-1, stearoyl-CoA desaturase-1; MTTP, microsomal triglyceride transfer protein.

Lipid metabolism involves lipolysis, lipogenesis and lipid transport (15). Certain key genes involved in lipid metabolism were detected in the present study. The mRNA expression levels of the lipolytic genes carnitine palmitoyltransferase 1 (CPT1) and adipose triglyceride lipase (ATGL) were significantly decreased in the livers of the HFD group and increased in the livers of the HFD + RHP_150_ group (P<0.01; Fig. 4A), whereas no significant alteration was observed in hormone-sensitive lipase (HSL) in HFD rats compared with RD rats (P>0.05). No significant changes in HSL transcription were observed following RHP administration to HFD rats (P>0.05). Conversely, the expression levels of fatty acid synthase (FASN) and stearoyl-coenzyme A desaturase 1 (SCD-1), two important lipogenic enzymes required for lipid synthesis, were significantly increased in the HFD group (P<0.01; Fig. 4B). In addition, administration of RHP reversed these increased levels of expression (P<0.01). Expression of the lipid transporter microsomal triglyceride transfer protein (MTTP) was also assessed. MTTP expression was downregulated in the HFD group (P<0.05; Fig. 4C) but no significant change in expression following RHP administration was identified (P>0.05).

These results demonstrated that RHP affected the expression of genes involved in lipid metabolism, with a dose-response association in the majority of the lipid metabolism gene expression in these two intervention groups.

## Discussion

Currently, NAFLD is the focus of significant scientific and clinical studies. In previous decades, several advances have been made in medical treatments of NAFLD. However, significant therapeutic success in clinical studies of NAFLD remains to be accomplished. To date, although lifestyle interventions remain the most effective method to control and remedy NAFLD in affected patients, pharmaceutical treatment is required to strengthen the therapeutic effects of lifestyle modifications (16–18).

Several pharmaceutical interventions have been used in present clinical NAFLD treatments, including antioxidants (4), insulin sensitizers (metformin and thiazolidinediones) (19), lipid-lowering drugs (20), angiotensin II receptor antagonists (irbesartan and losartan) (21,22) and other drugs, including ursodeoxycholic acid (23) and L-carnitine (24). However, there remains no definitive treatment for NAFLD as its pathology remains to be elucidated (5).

RHP is the major component of RH, a traditional Chinese medicine, and reportedly possesses several pharmacological activities. The present study demonstrated that RHP may be important in improving NAFLD by regulating adipose lipogenesis and lipolysis.

AMPK is a serine/threonine protein kinase that acts as a hepatocyte energy sensor, thereby regulating a wide variety of pathways involved in glucose and lipid metabolism (25). The present study demonstrated that RHP treatment decreased AMPK-α phosphorylation. The downstream target ACC, which is phosphorylated and inactivated by AMPK, can directly regulate lipid metabolism (26). In the present study, p-ACC demonstrated a similar change to p-AMPK, suggesting that RHP-induced alterations in lipid oxidation were mediated via AMPK activation.

Furthermore, AMPK regulates hepatic lipid metabolism by mediating the mRNA expression and transcriptional activity of PPARa and SREBP-1c, which regulate lipogenic and lipolytic gene expression, respectively (27,28). PPARα is a nuclear transcription factor that regulates lipid metabolism and fatty acid oxidation target genes (29). SREBP-1c is a major transcriptional regulator that controls the expression of key lipogenic enzymes to facilitate hepatic lipogenesis (30). In the present study, RHP treatment increased the expression of PPARα and decreased the expression of SREBP-1c compared with the HFD group. RHP treatment also promoted the expression of lipolytic genes and inhibited the expression of lipogenic genes in the liver, suggesting that RHP treatment may enhance hepatic fatty acid oxidation and suppress hepatic TG biosynthesis.

HSL is another catabolic rate-limiting steatolysis enzyme and is associated with TG metabolism (31). The present study demonstrated no significant change in the expression of HSL and it was hypothesized that administration of RHP may not affect all lipid metabolism genes.

In conclusion, the present study demonstrated that RHP treatment is effective at alleviating NAFLD in rats and its mechanism of action may be associated with AMPK activation and the regulation of genes involved in lipid metabolism.

## Figures and Tables

**Table I:** Nutritional values of rat diets.

Component	Regular diet	High-fat diet
g/100 g wet matter		
Moisture	9.20	5.12
Protein	22.10	12.31
Fat	5.28	2.94
Ash	5.20	2.90
Fibre	4.12	2.29
Carbohydrate	54.10	30.13
Lard	-	17.80
Saccharose	-	11.30
Casein	-	11.20
Gunk	-	2.00
Maltodextrin	-	2.00
kcal/100 g wet matter	352.00	444.70
% of total energy		
Protein	25.7	20.00
Fat	13.8	42.00
Carbohydrate	60.5	38.00

**Table II:** Primers used for semi-qPCR and qPCR.

Gene	Primer sequence	Product (bp)
IL-1β	Forward: 5′-CCTCTGTGACTCGTGGGATG-3′ Reverse: 5′-GGGTGTGCCGTCTTTCATCA-3′	277
TNF-α	Forward: 5′-TGAACTTCGGGGTGATCGGT-3′ Reverse: 5′-CTCCTCCGCTTGGTGGTTTG-3′	158
PPARα	Forward: 5′-AGACACCCTCTCTCCAGCTT-3′ Reverse: 5′-ACGCCAGCTTTAGCCGAATA-3′	200
SREBP-1c	Forward: 5′-GCCGAGGTGTGCGAAATG-3′ Reverse: 5′-GCACGGACGGGTACATCTT-3′	292
CPT-1	Forward: 5′-CCCTAAGCCCACAAGGCTAC-3′ Reverse: 5′-TCTCTGTCCTCCCTTCTCGG-3′	275
ATGL	Forward: 5′-TCCTCGGGGTCTACCACATT-3′ Reverse: 5′-AATCAGCAGGCAGGGTCTTC-3′	267
HSL	Forward: 5′-CTAGCCATACAGCAGCCCTC-3′ Reverse: 5′-ATGCTGTGTGAGAATGCCGA-3′	269
FASN	Forward: 5′-AGACGATGACAGGAGGTGGA-3′ Reverse: 5′-GAGTGAGGCCGGGTTGATAC-3′	199
SCD-1	Forward: 5′-ACCTTGCTCTGGGGGATATT-3′ Reverse: 5′-TTCCGCCCTTCTCTTTGACA-3′	298
MTTP	Forward: 5′-TCTGTGGTACCGCGAGTCTA-3′ Reverse: 5′-GGGTACTGGGAGAACTGCAC-3′	165
β-actin	Forward: 5′-CCCGCGAGTACAACCTTCTTG-3′ Reverse: 5′-ACCCATACCCACCATCACAC-3′	206

qPCR, quantitative polymerase chain reaction; IL-1β, interleukin-1β; TNF-α, tumor necrosis factor-α; PPARα, peroxisome proliferator-activated receptor α; SREBP-1c, sterol regulatory element binding protein-1c; CPT1, carnitine palmitoyltransferase I; ATGL, adipose triglyceride lipase; HSL, hormone-sensitive lipase; FASN, fatty acid synthase; SCD-1, stearoyl-CoA desaturase-1; MTTP, microsomal triglyceride transfer protein; bp, base pairs.
